# Characteristics and Drivers of High-Altitude Ladybird Flight: Insights from Vertical-Looking Entomological Radar

**DOI:** 10.1371/journal.pone.0082278

**Published:** 2013-12-18

**Authors:** Daniel L. Jeffries, Jason Chapman, Helen E. Roy, Stuart Humphries, Richard Harrington, Peter M. J. Brown, Lori-J. Lawson Handley

**Affiliations:** 1 Department of Biological Sciences, University of Hull, Hull, Humberside, United Kingdom; 2 Rothamsted Research, Harpenden, Hertfordshire, United Kingdom; 3 Environment and Sustainability Institute, University of Exeter, Penryn, Cornwall, United Kingdom; 4 NERC Centre for Ecology & Hydrology, Wallingford, Oxfordshire, United Kingdom; 5 Department of Life Sciences, Anglia Ruskin University, Cambridge, United Kingdom; Lund University, Sweden

## Abstract

Understanding the characteristics and drivers of dispersal is crucial for predicting population dynamics, particularly in range-shifting species. Studying long-distance dispersal in insects is challenging, but recent advances in entomological radar offer unique insights. We analysed 10 years of radar data collected at Rothamsted Research, U.K., to investigate characteristics (altitude, speed, seasonal and annual trends) and drivers (aphid abundance, air temperature, wind speed and rainfall) of high-altitude flight of the two most abundant U.K. ladybird species (native *Coccinella septempunctata* and invasive *Harmonia axyridis*). These species cannot be distinguished in the radar data since their reflectivity signals overlap, and they were therefore analysed together. However, their signals do not overlap with other, abundant insects so we are confident they constitute the overwhelming majority of the analysed data. The target species were detected up to ∼1100 m above ground level, where displacement speeds of up to ∼60 km/h were recorded, however most ladybirds were found between ∼150 and 500 m, and had a mean displacement of 30 km/h. Average flight time was estimated, using tethered flight experiments, to be 36.5 minutes, but flights of up to two hours were observed. Ladybirds are therefore potentially able to travel 18 km in a “typical” high-altitude flight, but up to 120 km if flying at higher altitudes, indicating a high capacity for long-distance dispersal. There were strong seasonal trends in ladybird abundance, with peaks corresponding to the highest temperatures of mid-summer, and warm air temperature was the key driver of ladybird flight. Climatic warming may therefore increase the potential for long-distance dispersal in these species. Low aphid abundance was a second significant factor, highlighting the important role of aphid population dynamics in ladybird dispersal. This research illustrates the utility of radar for studying high-altitude insect flight and has important implications for predicting long-distance dispersal.

## Introduction

An estimated three billion insects fly through a 1 km^2^ ‘window’ of sky in England during a typical summer month [1]. While a substantial proportion of these insects are beneficial and provide essential ecosystem services, others are pests that pose a potential threat to biodiversity, the economy and human health. Knowledge of the characteristics (e.g. altitude and displacement speed, seasonal and annual trends) and drivers (e.g. prey abundance, environmental factors) of insect flight is crucial for estimating long-distance dispersal capability, predicting the dynamics, persistence and spread of insect populations, and has important applications in pest management and conservation [2]–[6]. This knowledge is particularly important in the case of invasive alien species (IAS) and those undergoing range shifts in response to global warming [1]–[7], since higher temperatures have been shown to drive increased migration in certain insects [8]. Global warming could therefore increase the frequency and distance of migration, which, in the case of pest species, presents considerable challenges for conservation of native biodiversity and for sustainable agriculture.

Ladybirds (Coleoptera: Coccinellidae) are the most abundant aphid predators in cereal crops worldwide, and are important biological control agents of aphids and coccids [9]. However, use of certain species in biological control has contributed to their status as IAS in many parts of the world (see [6] and [10] for recent reviews). For example, *Coccinella septempunctata* was introduced to North America for biological control in the 1970s and rapidly spread across the continent, gaining IAS status in the 1990s (reviewed in [1], [10]). Soon afterwards (1988), another coccinellid, *Harmonia axyridis*, was found to be established and quickly spreading in North America [6]. Since 1988, *H. axyridis* has established in at least 38 countries on four continents, with spread rates estimated up to 500 km/year [6]. This rapid spread suggests considerable long-distance dispersal capability of this species, but estimates are confounded by accidental transport by humans [6]. Studying the dispersal capability of *H. axyridis* is particularly important given that this species has been linked to declines in indigenous ladybirds and is thought to present a threat to native biodiversity [11], [12].

Knowledge of the key characteristics of ladybird dispersal; specifically flight altitude, displacement speed, and seasonal and annual patterns, is crucial to understanding and predicting long-distance dispersal. Since wind speed increases with altitude, species that use high-altitude wind currents have greater long-distance dispersal potential than those that fly only a few metres above ground level (AGL), where wind currents are negligible [13]. Flight altitude is therefore a key determinant of dispersal potential. Our understanding of insect seasonal phenology tends to be drawn from ground-level observations, but it is unclear whether this accurately reflects insect density and population dynamics. For instance, the number of individual records of *H. axyridis* from field observations across the UK peaks in October, corresponding to the period when individuals are migrating to overwintering sites [14], but whether this corresponds to a peak in actual abundance is unclear. An important question is whether the potential for long-distance dispersal is greater during the migration to or from overwintering sites or during the summer months, when meteorological conditions may be more favourable for flight.

Another key question is whether dispersal is driven mainly by biotic or environmental cues, or by a combination of both. This is likely to vary depending on the scale of dispersal, as there are potentially different underlying causes for short and long-distance dispersal. Ladybird flight over short distances (classified as <2 m, and referred to as “trivial” or “appetitive flight” by [15]) is considered to be for foraging or oviposition, whereas flight over longer distances (>2 m), is thought to be in response to prey shortage or migration to or from over-wintering sites, which has an important physiological basis [15]. Several authors have reported a relationship between aphid density and ladybird emigration from a local foraging patch, with emigration rates increasing with decreasing patch quality [9], [16]–[23]. However, in some cases, even when aphids are abundant, a significant proportion of coccinellid adults disperse [24], [25], and the link between ladybird dispersal and local aphid density often appears to be relatively weak [26]. Environmental variables such as ambient temperature, wind speed and rainfall, may be equally or even more important than prey density in triggering dispersal. Elliott [27] found that aphid population density and ambient temperature were the main drivers of short-distance flights in three aphidophagous coccinellids, but that aphid density had no influence on longer flights. This is surprising given that one of the motivations for flight over longer distances is thought to be a response to food shortage [27]. Ambient temperature has a strong influence on insect metabolism and is considered an important predictor of insect flight [28]. Temperature is already known to act as a cue for migration to over-wintering sites in some coccinellids, including *H. axyridis* [29], and accounted for most of the variation in emigration rates of *Coccinella californica* from experimental plots of alfalfa and oats [24]. It is therefore likely to play an important role in long-distance dispersal, particularly if coccinellids are flying at high altitudes, where ambient temperature is much lower than at ground level. Wind speed is thought to be either facilitative or inhibitory in insect flight depending on its magnitude, and is potentially important in long-distance dispersal [6], although its impact on short-distance dispersal in coccinellids appears to be negligible [27]. Rainfall probably inhibits both short and long-distance dispersal, and to our knowledge this has not yet been investigated.

Both *H. axyridis* and *C. septempunctata* are considered to be strong, active fliers with high dispersal ability, at least over short distances [16], [18], [19], but studying dispersal over longer distances has been hampered by the difficulty of tracking the insects in the field. Conclusions have therefore been limited to assumptions inferred from ground level observations of walking [21], [30], [31] or short-distance flights [21], [24], [27], [32]–[34], or indirect evidence for long-distance flights (e.g. from malaise-traps, [35] or mark-release-recapture experiments, [21], [36]). To our knowledge, only one study has so far attempted to directly study coccinellids migrating at high altitudes. Using traps on aircraft, Hagen [37] sampled *Hippodamia convergens* flying to and from migration sites, between approximately 1000 and 1650 metres above ground level. However only 24 beetles were sampled in 15 aerial surveys. In recent years, a new generation of vertical-looking entomological radars (VLR) have made it possible to identify and record large insects flying at altitudes ∼150–1200 m AGL, providing quantitative estimates of insect aerial density, diversity and biomass, and unique insights into insect population dynamics and the characteristics and drivers of high-altitude [38]–[42] flight. Here, we analyse VLR data collected at Rothamsted Research, Harpenden, U.K. between May-October 2000–2010, together with meteorological and aphid suction trap data, and perform simple experiments to investigate high-altitude flight in *C. septempunctata* and *H. axyridis*. Specifically, we investigate 1) flight characteristics in relation to altitude, duration and speed, and seasonal and annual trends, and 2) the potential role of biotic (aphid abundance) and environmental variables (temperature, wind speed and rainfall) as drivers of high-altitude flight. We use time series analyses to investigate trends in ladybird abundance in relation to year, season, aphid abundance and environmental variables, and perform a combination of linear modelling techniques to identify the main driver(s) of high-altitude flight.

## Materials and Methods

### Vertical-looking radar (VLR) data and target species identification

Flight data for *H. axyridis* and *C. septempunctata* were obtained from vertical-looking entomological radar (VLR) data collected at Rothamsted Research in Harpenden (Hertfordshire), U.K. Radar equipment, mode of operation, and analysis capabilities have been previously described [1], [43]–[46]. In brief, the radar detects insects passing through a nutating, vertical beam, within 15 altitude bands located between 150 and 1189 m AGL [1] (and see Supporting Information Text S1). *H. axyridis* and *C. septempunctata* of both sexes were field-caught, weighed, and the two principal radar back-scattering terms were estimated from laboratory back-scattering measurements [47]. Selection criteria for filtering ladybird records from the VLR data were then based on expected body mass and back-scattering ratios for large ladybirds ([1], [43], [47] and Supporting Information Text S1). Mass and shape are considered species diagnostic characteristics [47], but species identification with the VLR cannot be performed with complete confidence, as there may be additional species from which a minority of individuals fall within the mass and shape range of the target species. *H. axyridis* and *C. septempunctata* have characteristically small body axis ratios relative to other insects, including many other coccinellids (Chapman et al., unpublished data). Coccinellids such as *Halyzia 16-guttata*, *Anatis ocellata*, *Harmonia 4-punctata, Myzia oblongoguttata*, *Coccinella magnifica* and *Henosepilachna argus* may overlap with the mass and shape ranges used to identify our target species, however, these species are far less common than either *C. septempunctata* or *H. axyridis*. Indeed, based on a very large long term UK and Ireland dataset [14], the two target species represent 87.1% of the total records for the eight coccinellid species listed above (Table S1). Moreover, our extensive experience of capturing high-flying insects in our aerial netting platform (at 200–250 m above the ground), gained from extended collection periods between 1999 and 2007 [7], [41] indicate that the only abundant day-flying insects with “ladybird-like” body shape flying at these altitudes, are indeed ladybirds. During this work, 91% of total ladybirds sampled were *C. septempunctata.* Based on this combined evidence, we are confident that the overwhelming majority of data points correspond to the two target species, *H. axyridis* and *C. septempunctata*. However, unfortunately, the large overlap between the mass and shape of these target species means it is impossible to distinguish between them. The VLR data set was filtered to extract records collected between 09:00 and 15:00 from May to October 2000–2010. The months between November and April were not analysed since records of ladybirds (and indeed most other insects) are negligible in the VLR data during this period. Aerial density (AD), an estimate of the target insect abundance standardized by the potential atmospheric sampling volume, was estimated for *H. axyridis* and *C. septempunctata* from the VLR data using an established protocol [1], [43].

### Characteristics and drivers of high-altitude flight

Aerial density and displacement speed (i.e. speed relative to the ground) of radar targets identified as large ladybirds were estimated at each of the 15 altitude bands using the filtered VLR database. Potential duration and distance of flight were investigated using a combination of displacement speed data obtained from the VLR, and flight duration data from tethered flight experiments in a custom-built flight simulator. Full details of the tethered flight experiments are provided in the Supporting Information Text S1, but briefly, 20 *H. axyridis* were tethered (individually) to the inside of a 1 m^3^ Perspex cube using fine fishing line, and their flight activity video-recorded over a 2-hour period. Video footage was then analysed to determine the mean and maximum time spent in active flight during the 2-hour period.

Monthly averages for each variable (aerial density, number of records, temperature, wind speed, rainfall and aphid abundance) were calculated for May to October 2000–2010. Temperature, wind speed and rainfall data were obtained for Rothamsted from the U.K Met Office Unified Model [48]. Outputs were extracted for each hour of each day encompassing the period for which VLR data were filtered. Values at 15 altitudes corresponding to the 15 VLR range gates were produced for each hour, providing an altitudinal and temporal profile for temperature and wind speed at the location of the Rothamsted Research VLR. Aphid abundance data were obtained from the Rothamsted Insect Survey (RIS) Aphid Bulletin (http://www.rothamsted.ac.uk/insect-survey/STAphidBulletin.php). Aphid data were collected in the same location as the VLR using a 12.2 m high suction trap, which samples 0.75 m^3^ of air/s [49]. Suction trap data are reliable proxies for estimates of aphid population size for the immediate area around catch sites [50]. Since our target coccinellids are both generalist predators, we included records for all 21 aphid species published by the RIS Aphid Bulletin during the study period in the analysis (see Table S2 for species list). Although the 21 species recorded in the bulletin represent a small fraction of the total number of aphid species present in the UK, they are the numerically dominant species, and account for approximately 87% of total aphid numbers (Table S2).

All statistical analyses were performed in R v2.15.1 [51]. First, we performed time series and seasonal decomposition analyses [52] to investigate seasonal, annual and overall trends for ladybird aerial density, temperature, wind speed, rainfall and aphid abundance. Linear trends in the time series were then investigated using linear regression, modified for time series data [52]. The relationship between aerial density and the explanatory variables was explored by cross-correlation plots of the auto-correlation function (ACF) and partial ACF (See Supporting Information Text S1 for further details).

Second, the effects of aphid abundance, temperature, wind speed, and rainfall on ladybird aerial density were investigated more formally, using statistical modelling, in order to identify the main driver(s) of ladybird flight. We first used standard linear regression to investigate the relationship between aerial density and each explanatory variable. Next, because of the violation of assumptions for linear regression, discussed below, we used Generalised Linear Models (GLMs) and Generalised Least Squares (GLS) models to identify the main driver(s) of high-altitude flight. Full details of data exploration and model validation are given in the Supporting Information Text S1. Briefly, we tested the assumptions of normality, homogeneity of variance, no colinearity between explanatory variables, no overdispersion and no autocorrelation in the time series. Normality and homogeneity assumptions were violated for aerial density, aphid abundance and rainfall, and these variables were therefore log transformed for all analyses. Evidence of colinearity was found between temperature and wind speed and between wind speed and rainfall (Figures S4 and S5 and Table S5), therefore wind speed was dropped from the full models (referred to hereon as the “partial model”), and results for full and partial models compared. No extreme outliers were detected in the data; however, plotting residuals and performing standard Poisson regressions (i.e. a GLM with a Poisson distribution and log link function) indicated that overdispersion might be a problem in the dataset. Quasi-Poisson GLMs (qpGLMs) were therefore performed to estimate and account for the dispersion parameter, *ρ*, in the models. Auto-correlation was investigated by examining the ACF value at different time lags in auto-correlation plots of the residuals obtained for selected models. We detected marginally significant positive auto-correlation between the same month in different years and negative auto-correlation at 15 and 27-month intervals (e.g. between May and August, or June and September of different years, Figure S6) and therefore examined its effect further using GLS models with and without auto-correlation, as detailed below, to examine the impact of temporal auto-correlation on the results.

In both the qpGLM and GLS analyses, we compared full (i.e. including aphid abundance, temperature, wind speed, and rainfall as explanatory variables), and partial (excluding wind speed) models to examine the effects of significant colinearity between wind speed and other environmental variables. The optimal minimal model (i.e. containing only significant explanatory variables) was found by dropping one explanatory variable in turn and each time applying an analysis of deviance (*F*) test. To examine the impact of temporal auto-correlation using GLS modelling, we first performed model simplification, starting from both the full and partial models, which is equivalent to a standard multiple linear regression. Next, we added date (month or year) as an extra explanatory variable in the model, and tested for its effects. Finally, we extended the full and partial models (excluding date) to allow the residuals to have a temporal pattern, which relaxes the independence assumption [53]. We imposed an AR-1 auto-correlation structure (i.e. an autoregressive model of order 1), which models the residuals and noise at time *s* as a function of the residuals of time *s* – 1 [53]. GLS models were fitted using the Residual Maximum Likelihood (REML), and the retained models compared by their Akaike and Bayesian Information Criteria (AIC and BIC respectively) and log likelihood scores.

## Results

### Identification of target species and flight characteristics

**Figure 1:**
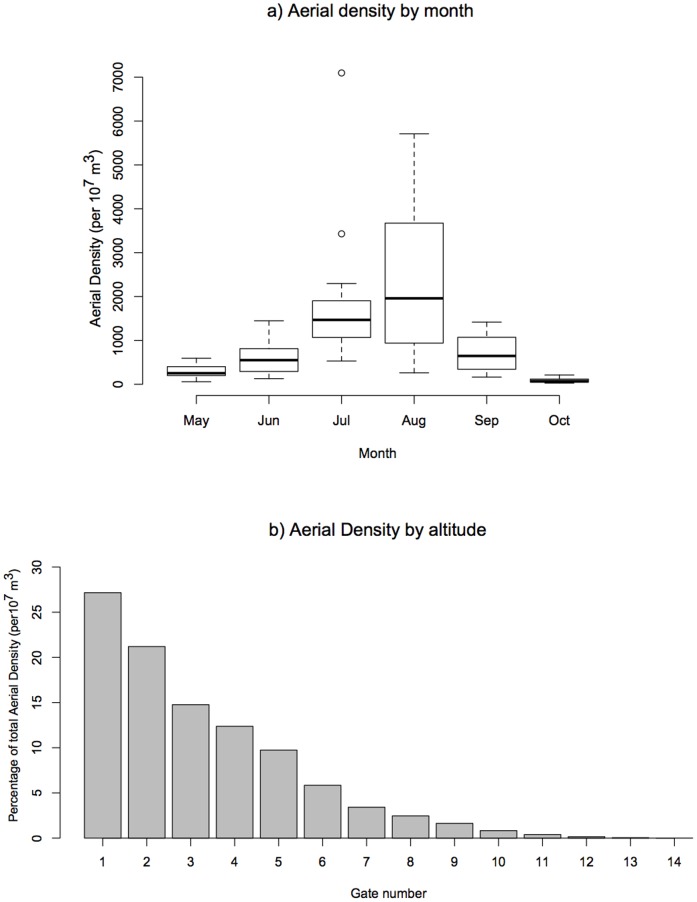
Aerial density summarized by a) month and b) altitude for 2000–2010. Figure 1a Box plot for ladybird aerial density summarized by month. Boxes correspond to the 25^th^ and 75^th^ percentile, horizontal bars within boxes to means, and whiskers to maximum values or 1.5 times the interquartile range (when there are outliers present, represented by open circles). For boxplots of aerial density by year, or number of target species records in the VLR database, see Figure S1. Figure 1b Barplot of percentage total aerial density by altitude. The majority (roughly 85%) of ladybirds were detected in the first 5 range gates. Gate numbers correspond to the following altitudes (AGL): 1: 150–195; 2: 221–266; 3: 292–337; 4: 363–408; 5: 434–479; 6: 505–550; 7: 576–621; 8: 647–692; 9: 718–763; 10: 789–834; 11: 860–905; 12: 931–976; 13: 1002–1047; 14: 1073–1118; 15: 1144–1189.

A total of 8935 large ladybird-type targets (i.e. presumed *C. septempunctata* and *H. axyridis*) were detected in the VLR data during the study period (see Data S1). There was an almost perfect linear relationship between the number of records and aerial density (by year, *R^2^_adj_*  = 0.911, *F_1,9_* = 103.4, *P*<0.001), therefore we just present the results for aerial density here (see Table S3 for a full breakdown of the sum and mean number of records and aerial density by year and month). The highest aerial density was in 2006 (sum  = 14317, monthly mean 2386, Standard Deviation, S.D.  = 2969), with the lowest in 2000 (sum  = 3014, monthly mean 502, S.D.  = 587). Aerial density by month followed a normal distribution with a peak in August (sum  = 26751, annual mean  = 2432, S.D.  = 1924), and lowest values in October (sum  = 788, annual mean  = 72, S.D.  = 64) and May (sum  = 2767, annual mean  = 252, S.D. 180, Figure 1, Figure S1, Table S3).

The target species were detected in gate numbers 1–14 of the VLR (corresponding to altitudes between 150 and 1118 m AGL, Figure 1b and Table S4). Aerial density follows an exponential distribution (Figure 1b), with the greatest number of detections in first range gate (150–195 m AGL, AD sum  = 18145, mean  = 13, S.D.  = 7) and the fewest in the fourteenth range gate (corresponding to 1073–1118 m AGL, aerial density sum  = 4.0, mean  = 2.0, S.D.  = 0.1). The majority (85.2%) of ladybirds were detected in the first five range gates, indicating that most ladybird flight within the detected range occurred roughly between ∼150 and 500 m AGL (Table S4).

Mean displacement speed of the target species ranged from 8.5 m/s (S.D.  = 4.2) for gate 1 to 16.4 m/s (S.D.  = 1.3) for gate 14 (Table S4). The mean displacement speed of the target species in the first 5 gates (corresponding to 85% of the detections, as noted above) was 8.4 m/s (S.D.  = 0.3). Tethered flight experiments for *H. axyridis* produced a mean uninterrupted flight duration of 36.5 (±35.5) minutes, with a maximum flight duration of 2 hours, at which time flights were stopped.

### Time series analyses

#### Are there any seasonal, annual or overall trends in the time series?

**Figure 2:**
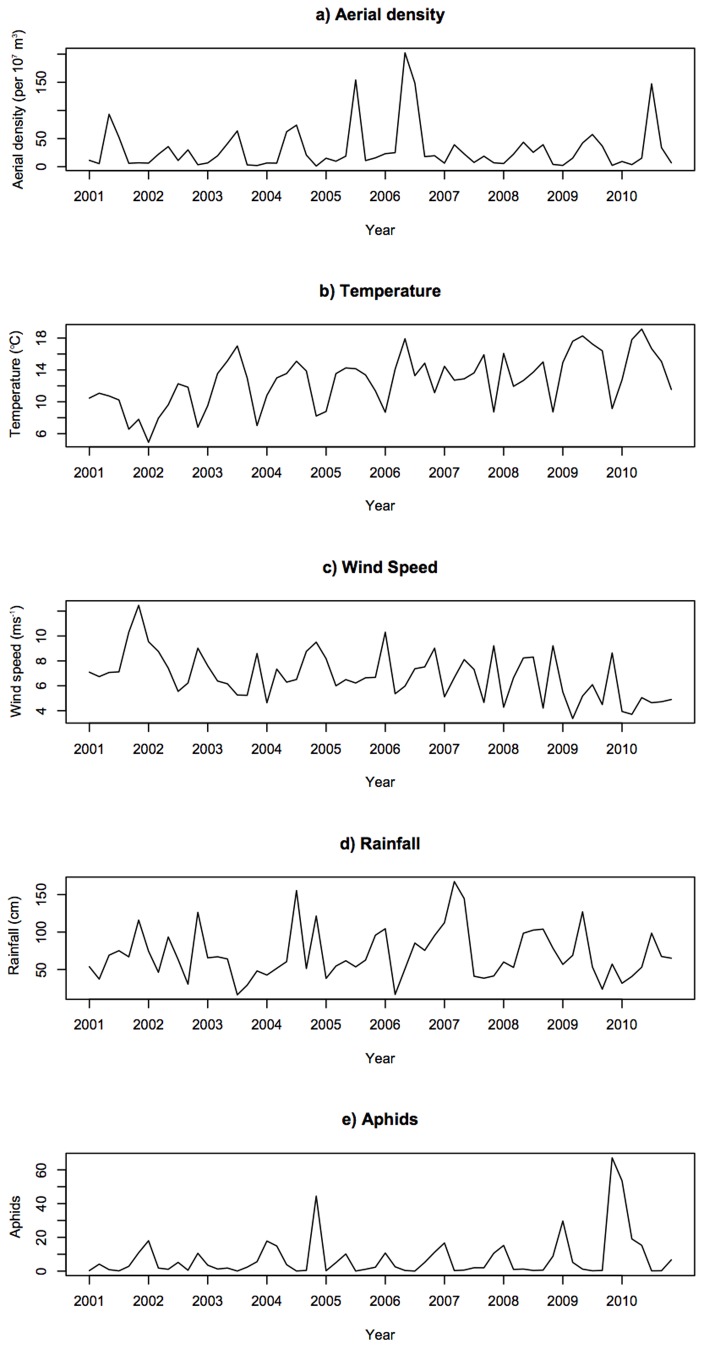
Time series plots for each variable. Results of time series analysis for aerial density and each explanatory variable for May to October 2000–2010. Note the correspondence between peaks in temperature and aerial density, and the lag between peaks in aerial density and aphid abundance.

Both basic time series plots (Figure 2) and seasonal decomposition plots (Figure S2) demonstrate clear seasonal peaks and troughs for ladybird aerial density and aphid abundance. Ladybird aerial density peaked during midsummer of 2005, 2006 and 2010, suggesting a positive relationship with temperature, whereas aphid numbers troughed in midsummer, and peaked late summer/early autumn of 2004 and 2010, and then in the spring of the following years. Peaks in aphid abundance therefore precede those for ladybird aerial density, and ladybird flight activity is at its highest when aphid numbers are at their lowest. However this association is complex. For example, high aphid abundance does not explain the peak in ladybird aerial density for 2006, since 2005 was a poor year for aphids.

Plots suggest linear trends for temperature and wind speed over the whole study period, and this was confirmed by linear regressions, which showed a significant increase in temperature (*R^2^_adj_*  = 0.287, *F*_1,58_  = 24.77, *P* = 0.000) and decrease in wind speed (*R^2^_adj_*  = 0.175, *F*_1,58_  = 13.49, *P* = 0.001) over the time series. Time series plots do not however indicate any linear trends for aerial density, rain or aphids. The time series plots also indicate potential relationships between explanatory variables, particularly a negative relationship between temperature and wind speed. Potential colinearity between explanatory variables was therefore investigated further during model validation (see below and Text S1)

#### Is there evidence of auto-correlation or partial auto-correlation in the time series?

Significant auto-correlation in the same month of each year was found for ladybird aerial density, temperature and wind speed, but not for rainfall or aphid abundance (Figure S3 a–e respectively). This was confirmed in the partial auto-correlations (Figure S3 f–j). Aerial density also exhibits significant partial auto-correlation at a lag of five months. For example, it will be high in October, if it was high in May (Figure S3 f). Aphids follow a similar pattern, but the partial ACF at a lag of five months is not quite significant (Figure S3 j).

#### Is there evidence of cross-correlation between ladybird aerial density and the explanatory variables?

**Figure 3:**
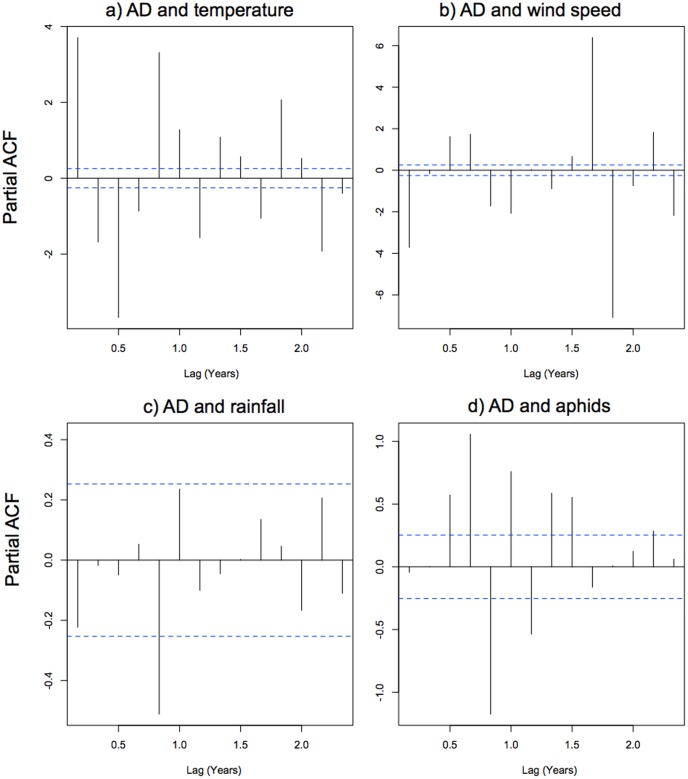
Partial auto-correlation plots for aerial density against the four explanatory variables. “ACF” is the auto-correlation function, and “AD” Aerial density. Peaks that cross the dotted blue lines are considered significant at the 5% level. All explanatory variables show at least some significant peaks suggesting some influence on aerial density, however patterns for temperature, wind speed and aphids are particularly strong (Figure 3 a, b and d).

We focus here only on results from partial auto-correlations since they are much more informative than auto-correlations for revealing relationships between variables as they account for correlation between successive points in the time series. All pair-wise cross correlations showed at least some significant peaks in the partial auto-correlation plots, suggesting influence of the explanatory variables on AD (Figure 3). However, patterns for temperature, wind speed and aphids are particularly strong (Figure 3 a, b and d). Partial auto-correlation is significant at each lag in the time series for AD versus temperature (Figure 3a) and wind speed (Figure 3b), and for the majority of lags in the time series of AD versus aphids (Fig 3e). This indicates that temperature, wind speed and aphid abundance are key drivers of ladybird aerial density. However, as mentioned above, wind speed and temperature are not independent. Indeed, the relationship between AD and wind speed shows the exact opposite trend to that for AD and temperature (i.e. a significant positive peak for aerial density versus temperature corresponds to a significant negative peak for aerial density versus wind speed, or vice versa), which indicates strong co-linearity between wind speed and temperature.

### Statistical models

#### What are the key driver(s) of high-altitude flight?

**Figure 4:**
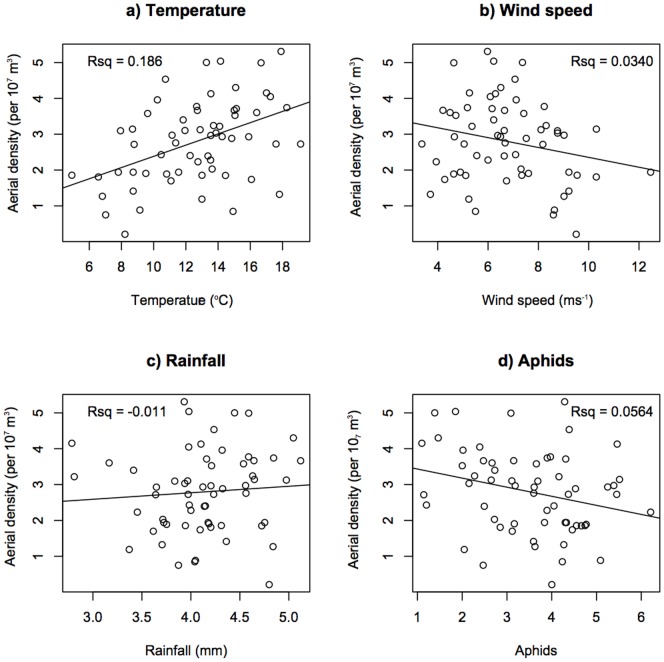
Linear regression of aerial density against all explanatory variables. Graphs show the relationship between monthly mean aerial density and each of the explanatory variables (also monthly means). Units for the explanatory variables are: temperature: °C, wind speed: m/s, rainfall: mm, aphids: absolute number counted in suction trap. Note aerial density, rainfall and number of aphids are not normally distributed and are therefore log transformed (see main text). “Rsq”  =  adjusted *R^2^* (*R^2^_adj_*). Temperature and aphids are both significant predictors of aerial density (see main text).

Significant relationships were identified between AD and both temperature and aphid abundance in the standard linear regressions (Figure 4 a and d). Aerial density increases with increasing temperature up to approximately 19°C (Figure 4 a), with temperature explaining approximately 19% of the variance in AD (adjusted *R* squared value, *R^2^_adj_*  = 0.186, *F_1,58_*  = 14.47, *P* = 0.000). There is a weak, but significant, negative relationship between aphid abundance and AD (Figure 4d), with aphids explaining approximately 6% of the variance (*R^2^_adj_*  = 0.056, *F_1,58_*  = 4.527, *P* = 0.038). No relationship was found between AD and either rainfall (*R^2^_adj_*  = −0.011, *F_1,58_*  = 0.358, *P* = 0.552, Figure 4c) or wind speed (*R^2^_adj_*  = 0.034, *F_1,58_*  = 3.080, *P* = 0.085, Figure 4b). The importance of both temperature and aphid abundance as predictors of aerial density were confirmed by our GLM and GLS analyses, as discussed below.

We found a strong, negative linear relationship between temperature and wind speed (correlation coefficient  = −0.8, *R^2^_adj_*  = 0.574, *F_1,58_*  = 80.470, *P* = 1.484×10^−12^) and a positive linear relationship between wind speed and rainfall (correlation coefficient  = 0.3, *R^2^_adj_*  = 0.101, *F_1,58_*  = 7.654, *P*  = 0.008, see Table S5). Partial models, excluding wind speed were therefore constructed and compared to full models to examine the effect of colinearity between wind speed and other environmental variables. The effects of removing wind speed from the full qpGLM and GLS models are presented in full in Text S1. Briefly, removing wind speed from the full models increased the level of significance for temperature in both analyses, but did not affect other explanatory variables. Removing wind speed also improved the GLS models slightly, as demonstrated by lower AIC, BIC and log likelihood scores in the partial compared to full models (Tables S6 and S7).

Temperature and aphid abundance were the only significant predictors of ladybird aerial density in the full and partial qpGLMs (Tables S6 and S7), and were therefore retained in the minimal (optimal) model (Table 1). Dropping temperature or aphid abundance from the models also resulted in a significant reduction in the explained deviance (Table 1). Although co-linearity between wind speed and other environmental variables was a potential concern, we found no evidence of an interaction between temperature and wind speed (*t* = −1.563, *P* = 0.125) or between rainfall and wind speed (*t* = −1.955, *P* = 0.057) in the full qpGLMs, suggesting wind speed has little influence on the outcome.

**Table 1:** Predictors of aerial density: Minimal (optimal) model.

Variable	qpGLM *t* (*P*)	qpGLM deviance (*F* and *P*)	GLS without auto-correlation *t (P)*	GLS with auto-correlation *t (P)*
Aphid abundance	−2.292 (0.026^+^)	23.750 (*F* = 4.982, *P* = 0.030^+^)	−2.273 (0.027^+^)	−2.349 (0.022^+^)
Temperature	3.847 (0.000^***^)	26.046 (*F* = 10.782, *P* = 0.002^*^)	3.879 (0.000^***^)	3.601 (0.001^**^)
AIC	n/a	n/a	185.729	184.958
BIC	n/a	n/a	193.901	195.173
Log likelihood	n/a	n/a	−88.865	−87.479

Tables S6 and S7 for results of full model including wind speed and partial model excluding wind speed, respectively). For the qpGLMs, both *t* statistic, and *F* statistic with corresponding and *P* values are given. The qpGLM dispersion parameter, ρ = 0.374, and residual deviance  = 22.932 on 57 degrees of freedom (df). Residual df  = 57 for each model. Note that the GLM deviance, *F* and *P* values correspond to the full model, where residual deviance is 21.777 on 55 df. GLS without autocorrelation is equivalent to standard multiple linear regression. Results for minimal qpGLMs and GLS models (See

*P* value codes: ^***^ *P*<0.000; ^**^ *P*<0.001; ^*^ *P*<0.01; ^+^ *P*<0.05.

Temperature and aphid abundance were also the only significant predictors of aerial density in full and partial GLS models (Tables S6 and S7) and were therefore retained in the minimal model (Table 1). Date (month or year) was not a significant predictor in the full or partial GLS models when added as an extra explanatory variable, and adding date increased the AIC, BIC and log likelihood scores. Adding an auto-correlation structure did not improve the full or partial GLS models, and did not change overall conclusions about the importance of explanatory variables. Adding an auto-correlation structure to the minimal GLS model slightly reduced the significance of temperature, but did not change the outcome for aphid abundance, nor improve the minimal model (Table 1).

Together, the linear regression, qpGLM and GLS results demonstrate that both temperature and aphid abundance are significant drivers of high-altitude ladybird flight, with temperature being the most important explanatory variable. The results are robust to different methods of analysis, and are essentially unaffected by confounding factors such as co-linearity and auto-correlation.

## Discussion

### Characteristics of high-altitude ladybird flight

Previous studies have been limited to studying coccinellids flying at or near ground level (e.g. [21], [24], [27], [30], [31], [33], [52]–[55]. In this study we aimed to enhance understanding of high-altitude flight for two economically and ecologically important coccinellids, both of which are considered IAS in large parts of the world. Data obtained from vertical-looking radar shows that ladybirds have a high propensity for high-altitude flight, with flight recorded up to 1118 m above ground level. However, the majority (85%) of ladybirds were found between 150 m (the lowest range gate of the VLR) and 479 m, perhaps due to decreasing air temperatures and energetic requirements of reaching higher altitudes.

Direct estimates of the long-distance dispersal capability of coccinellids are crucial for accurately predicting spread beyond native ranges, and could inform risk assessment. Indirect estimates of the spread of *H. axyridis* in its invasive range vary from 105 km/year in the U.K. [57] to 500 km/year in South Africa [58], but these estimates are influenced by anthropogenic factors and/or obtained from historical records, which may be incomplete [6]. Here we estimated the speed and potential distance of ladybirds flying at high altitudes, using a combination of data from tethered flight experiments and the VLR. The mean displacement speed of ladybirds detected flying through the VLR sampling volume ranged from 31 km/h at 150 m AGL to 59 km/h at 1500 m AGL, with the majority of ladybirds (85%, detected at altitudes of 150 – 479 m AGL) having a mean velocity of 30 km/h. Mean flight time for *H. axyridis* was 36.5 minutes in tethered flight experiments, which is similar to that found by Rankin & Rankin [59] in *Hippodamia convergens*, although some individuals continued flying for the two-hour duration of the experiment. Although it is impossible to say whether experimental estimates of flight time accurately reflect flight in the field, it suggests that on average (i.e. assuming speeds of 30 km/h for 36.5 minutes and that meteorological conditions are similar to those in this study), ladybirds can fly 18 km in a single flight. However, a few individuals, flying at very high altitudes and for longer periods (assuming 59 km/h for two hours), could potentially disperse almost 120 km in a single flight. Little similar data exists from other taxa for comparison, however moths and butterflies, also in the south of England, have been estimated to travel up to 400–700 km in a single 8 hour migratory flight [48], which is broadly in line with our estimates.

Time series analyses demonstrated clear seasonal cycles in ladybird flight, with peak aerial density corresponding to midsummer, and lowest aerial densities in May and October. Low aerial density in May corresponds to the period when *C. septempunctata* and *H. axyridis* adults are typically mating, supporting a previous study which demonstrated that the flight activity of *C. septempunctata* drops to near zero when breeding [60]. *C. septempunctata* and *H. axyridis* begin to fly to overwintering sites during October [9], and it may therefore seem surprising that no peak in aerial density was found during this month. In the U.K. however, overwintering sites are generally close to breeding sites, so high-altitude flight is not likely to be required. Moreover, generally cool temperatures and higher wind speeds at this time of year could limit opportunities for high-altitude flights, as was found in *C. septempunctata* in Northern Hungary [56]. It is, therefore, likely that flights to overwintering sites occur below 150 m AGL (the minimum detection threshold of the VLR), which matches increases in observations of *C. septempuncata* at 12.5 – 27 m AGL during migrations to diapause sites in Hungary [56]. Similar seasonal patterns to those found here have been found for *C. septempunctata* from U.K. Ladybird Survey records (phenogram based on 27,000 records collected over 1785×10 km squares between 1990 and 2010 [14]). However the U.K. Ladybird Survey phenogram for *H. axyridis* (based on 25,676 records collated from 1099×10 km squares between 2004 and 2010 [14]), provides a different picture, with a small peak in the number of records between June and July, and greatest numbers between October and November. This difference can be explained by the preference for *H. axyridis*, but not *C. septempunctata*, to overwinter inside buildings in very large numbers, making them highly conspicuous during autumn.

### Drivers of high-altitude ladybird flight

A complex but well-established link exists between aphidophagous coccinellids and the population dynamics of their prey [26], [61]–[63] and the density of adult ladybirds is often positively correlated with aphid density [9. 20, 64, 65]. Previous work has shown that ladybird emigration rate often decreases with increasing prey abundance ([19], [21], [22], [24], [27] and see [26] for review), but this pattern has not been confirmed for all aphidophagous coccinellids studied, including *H. axyridis* [22], and so far studies have focused on dispersal for foraging over short distances (but see [21]). We therefore aimed to answer the question: “can changes in aphid abundance explain high-altitude flight and long-distance dispersal in *C. septempunctata* and *H. axyridis*, or are environmental variables, most notably temperature, more important?” Time series analyses highlighted the association between ladybirds and their aphid prey, with peaks in aphid abundance (generally seen in autumn and spring) preceding those for ladybird aerial density (in midsummer), consistent with ladybirds dispersing at times of low aphid abundance. This was supported by a weak but significant, negative relationship between aphid suction trap catches and ladybird aerial density, with aphids explaining approximately 6% of the variation in aerial density. Aphid abundance (together with temperature, discussed below) was retained in all three of our statistical models, indicating significant association with peaks in ladybird flight. Aphid abundance therefore appears to be an important driver of ladybird dispersal. It should be noted however that only dispersing aphids are caught in the suction traps, but there is good evidence that suction trap catches are indicative of aphid numbers in the field [2], [50].

Although our results indicate that aphids clearly influence dispersal, they demonstrate that ambient temperature is a much more important driver of ladybird flight in the U.K. Time series analyses suggested a positive relationship between monthly temperature and ladybird aerial density, with peak measurements corresponding to high summer. This was confirmed with linear regressions, in which temperature (between 5 and 19°C) explained approximately 19% of the variation in aerial density. Moreover, temperature was retained, and highly significant in all of our statistical models. This is perhaps not surprising given that temperature has long been implicated as the single most important predictor of insect flight [28]. In addition, temperature has previously been shown to influence emigration rate from experimental alfalfa and oat fields in *Coccinella californica* [24] and ground level dispersal over short (<2 m) or longer (>2 m) distances in *Coleomegilla maculata, Hippodamia convergens*, and *Hippodamia tredecimpunctata* [27]. However its impact on high-altitude flight has been rarely investigated in any insect (but see [43], [45], [66]) and its effect is of critical importance given the increasing pressure from global warming, and the implications for increased long-distance dispersal ability of range-shifting species.

We predicted that wind speed and rainfall would also influence high-altitude ladybird flight, since wind speed is thought to be either facilitative or inhibitory in insect flight depending on its magnitude [6], and rainfall could be expected to have an inhibitory effect on flight. For example, [40] found that the nocturnal moth *Autographa gamma* actively chooses high-altitude wind jets and also compensates for cross-wind drift to facilitate long distance dispersal. However we found no effect of either variable, even after accounting for co-linearity between wind speed and temperature, and after removing temperature from statistical models. Note though, that our analysis was restricted to ground-level wind speed data, recorded by the U.K. Met Office. Although ground-level wind speed is a good proxy for wind speed at higher altitudes, the potential role of windborne dispersal at high altitudes should be investigated further in ladybirds.

In the current study we were only able to consider environmental variables and aphid abundance as potential drivers of dispersal. Other potential drivers include the physiological state of beetles at the onset and completion of diapause. Further study is needed to determine the relative importance of physiological compared to other biotic and abiotic cues.

### Conclusions and implications for long-distance dispersal

In conclusion, *C. septempunctata* and *H. axyridis*, have a high propensity for long-distance dispersal, having been detected flying over southern England at altitudes up to 1118 m AGL at speeds of up to 59 km/h. Temperature is a key driver of high-altitude ladybird flight at least in the U.K., and low aphid abundance is likely an important cue for long distance dispersal. Temperature may in fact partly explain the colonisation pattern of *H. axyridis* in the U.K. which, despite a rapid spread across southern England, has slowed dramatically since it reached the boundaries of the Cambrian and Pennine mountains [6]. It may be that colder temperatures in these mountains inhibit flight and therefore act as barriers to dispersal in the U.K. If global temperatures continue to increase, such barriers to dispersal may be compromised and this could potentially facilitate the spread of these species. Predictive models of range expansion in invasive species are needed to inform response strategies [67], and accuracy of these models will increase as the number of assumptions about an invader's biology decreases [68]. Insights into the characteristics and drivers of dispersal, as produced here, are therefore crucial if comprehensive predictive models are to be used for risk assessment and management of IAS.

## Supporting Information

Figure S1 **Boxplots of the number of target species VLR records and aerial density by Month (a and b) and Year (c and d).**
(TIFF)

Figure S2 **Decomposition of a) aerial density and b) aphid abundance time series into seasonal, trend and remainder components.** The seasonal component was estimated by taking the average per month.
(TIFF)

Figure S3 **Auto-correlation (a–e) and partial auto-correlation (f–j) plots.** “ACF” is the auto-correlation function. Peaks that cross the dotted blue lines are considered significant at the 5% level.
(TIFF)

Figure S4 **Pairplot of aerial density and explanatory variables.** The lower diagonal panels contain the absolute correlations, with the font size proportional to the value. The upper diagonal shows the pair-wise scatter plots with LOESS smoothing lines added. Note aerial density, rainfall and aphids are log transformed.
(TIFF)

Figure S5 **Relationship between explanatory variables.** Pair-wise linear regression between explanatory variables. Note rainfall and aphids are log transformed. “Rsq”  =  *R^2^_adj_* (adjusted *R^2^*).
(TIFF)

Figure S6 **Auto-correlation plot of residuals for the selected minimal model** (which includes the explanatory variables temperature and aphid abundance). “ACF” is the auto-correlation function. Peaks that cross the dotted blue lines are considered significant at the 5% level. The plot demonstrates marginally significant positive auto-correlation between the same month in different years, and negative auto-correlation at a lag of 9 and 15, which corresponds to 15 and 27-month intervals in real-time (e.g. between May and August, or June and September of different years).
(TIFF)

Table S1 **Length ranges and number of records for all large ladybird species in the U.K. ladybird survey between 1990 – 2010.**
(DOCX)

Table S2 **The proportion of the total number of aphid catches for all species detected by the Rothamsted Insect Survey (RIS) UK suction trap network that is accounted for by the 21 aphid species included in the RIS aphid bulletin.**
(DOCX)

Table S3 **Summary of the number of target species VLR records and aerial density by year and month.** The total number of target species records in the VLR database is 8935. “S.D.” Standard deviation.
(DOCX)

Table S4 **Summary of the number of target species VLR records, aerial density and displacement speed by altitude.**
(DOCX)

Table S5 **Relationship between explanatory variables.** Results of pair-wise linear regression exploring collinearity between explanatory variables. Upper diagonal  =  *R^2^_adj_* (adjusted *R^2^*), lower diagonal  =  *F* statistic and *P* value (in brackets). All *F* statistics are on 1 and 58 degrees of freedom. Significant relationships are highlighted in bold. Note rainfall & aphids are both transformed.
(DOCX)

Table S6 **Drivers of high-altitude flight**: **full model (including wind speed).** Results shown for qpGLMs and GLS are for full model containing all explanatory variables including wind speed. “qpGLM is quasi-Poisson full model. Both *t* statistic, and *F* statistic with corresponding and *P* values given. Dispersion parameter for qpGLM *ρ* = 0.369, and deviance 21.777 on 55 df. GLS without autocorrelation is equivalent to standard multiple linear regression. *P* value codes: ^***^ *P*<0.000; ^**^ *P*<0.001; ^*^ *P*<0.01; ^+^ *P*<0.05.
(DOCX)

Table S7 **Drivers of high-altitude flight**: **partial model (excluding wind speed).** Dispersion parameter for qpGLM *ρ* = 0.367, and deviance 22.123 on 56 df. *P* value codes: ^***^ *P*<0.000; ^**^ *P*<0.001; ^*^ *P*<0.01; ^+^ *P*<0.05.
(DOCX)

Text S1 **Supplementary Methods and Results.**
(DOCX)

Data S1 **Raw data file.** Final VLR data set filtered for *C. septempunctata* and *H. axyridis* like hits. For an explanation of sigma values please see Supplementary Text S1.
(XLSX)
