# Supplementary figures and images for: Characteristics and Drivers of High-Altitude Ladybird Flight: Insights from Vertical-Looking Entomological Radar
Source: PLoS One. 2013 Dec 18;8(12):e82278. doi: 10.1371/journal.pone.0082278 (PMC3867359; doi:10.1371/journal.pone.0082278)

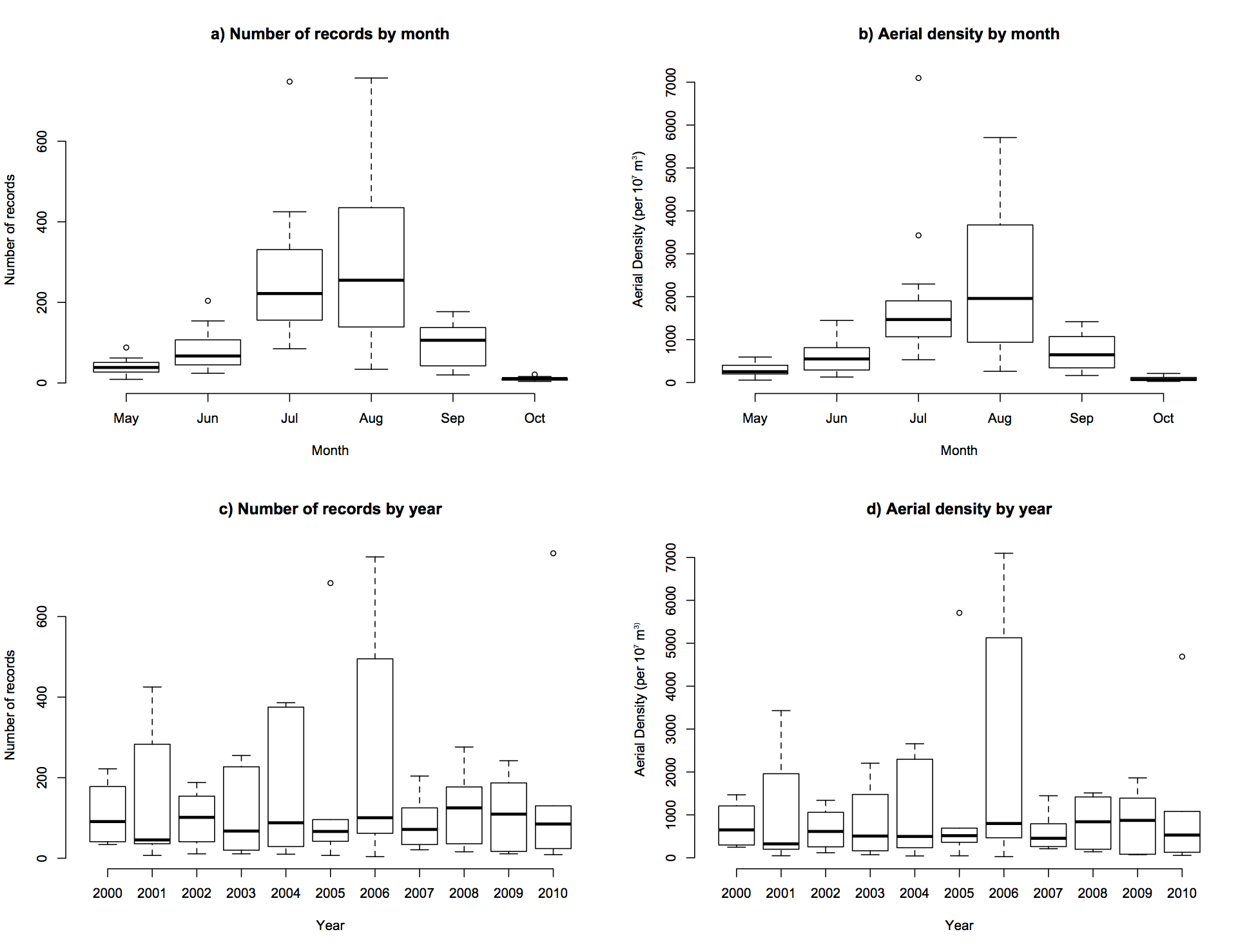

Supplement: Figure S1 — Boxplots of the number of target species VLR records and aerial density by Month (a and b) and Year (c and d). (TIFF) [file pone.0082278.s001.tiff]

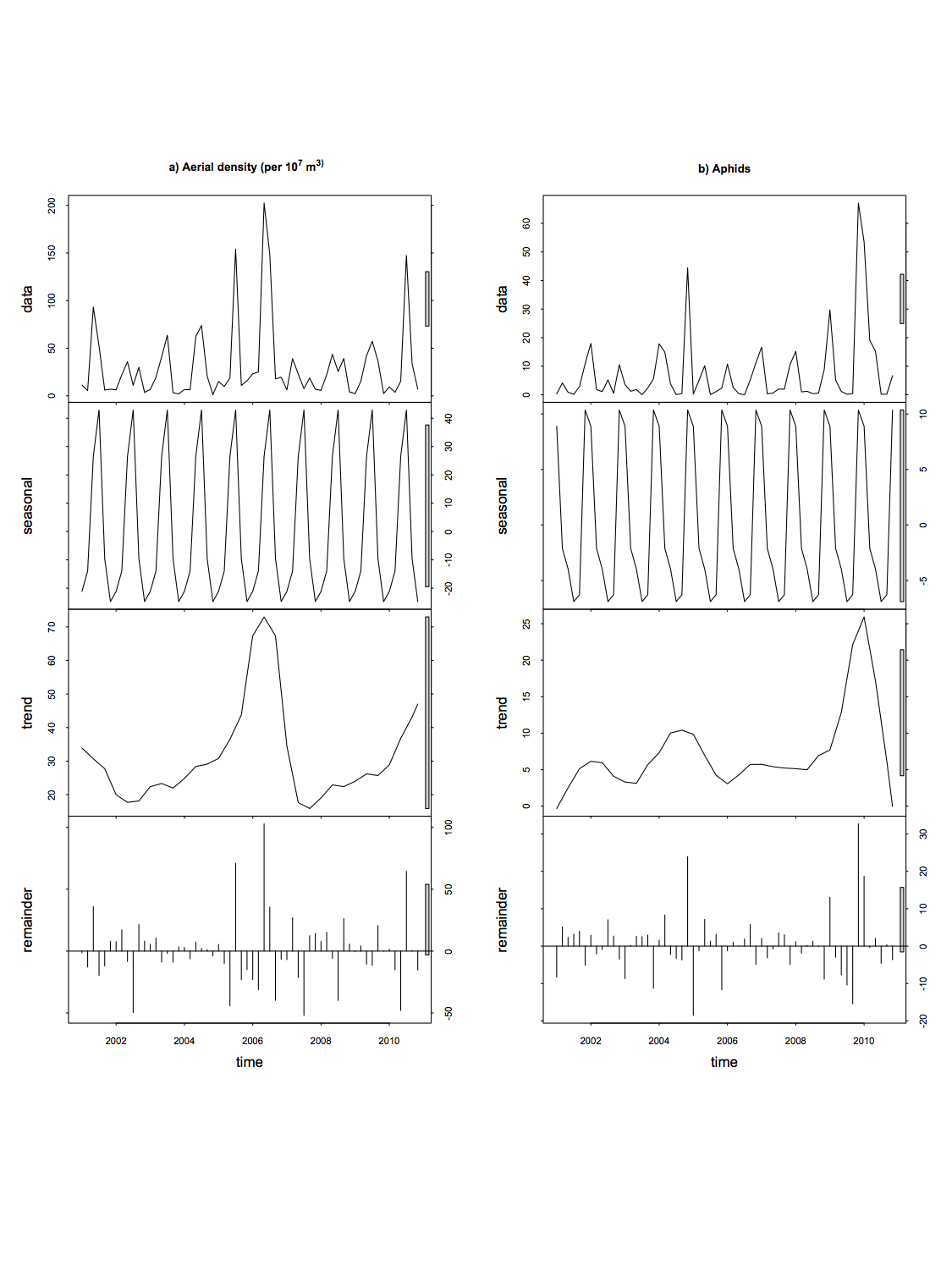

Supplement: Figure S2 — Decomposition of a) aerial density and b) aphid abundance time series into seasonal, trend and remainder components. The seasonal component was estimated by taking the average per month. (TIFF) [file pone.0082278.s002.tiff]

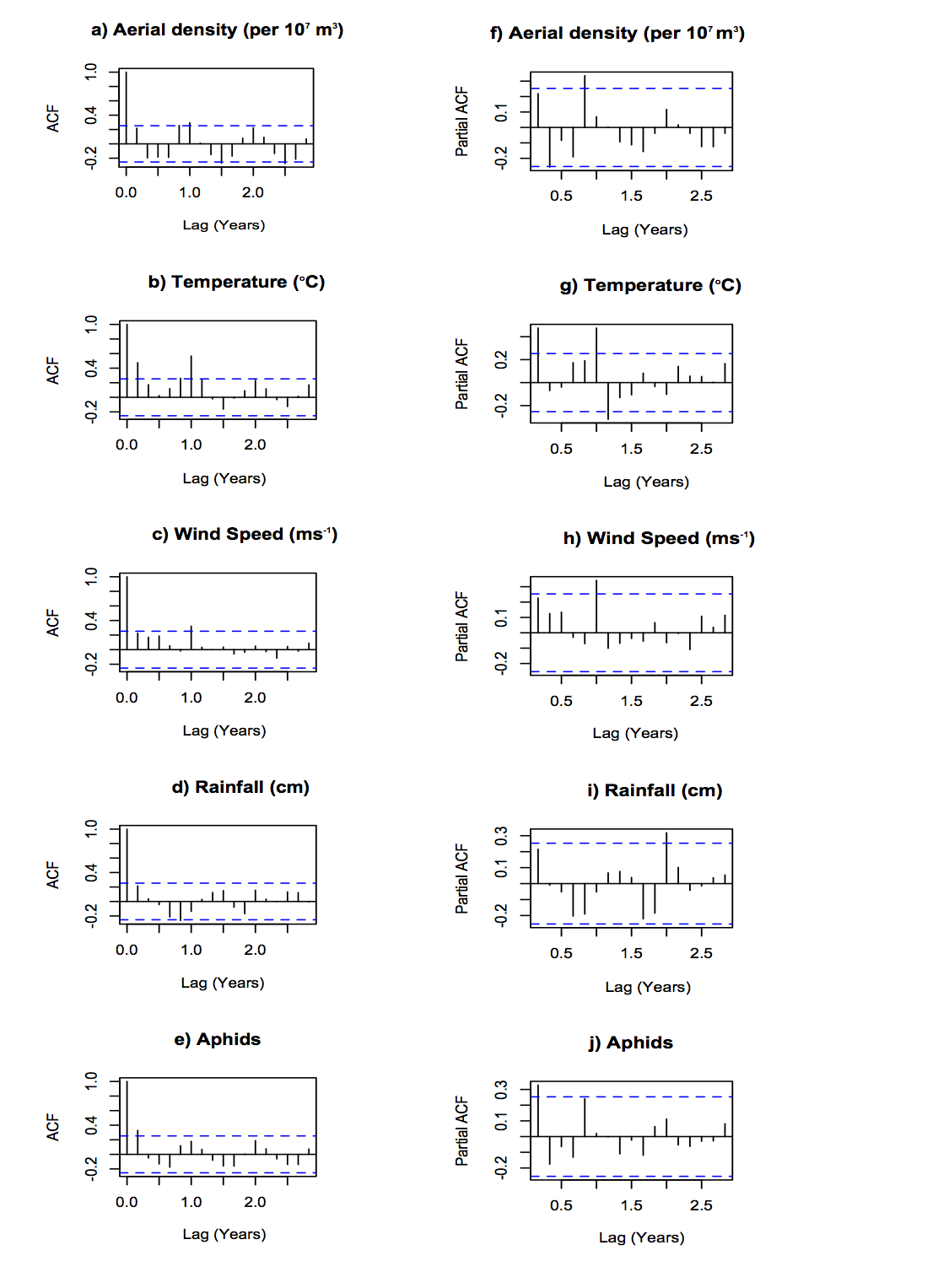

Supplement: Figure S3 — Auto-correlation (a–e) and partial auto-correlation (f–j) plots. “ACF” is the auto-correlation function. Peaks that cross the dotted blue lines are considered significant at the 5% level. (TIFF) [file pone.0082278.s003.tiff]

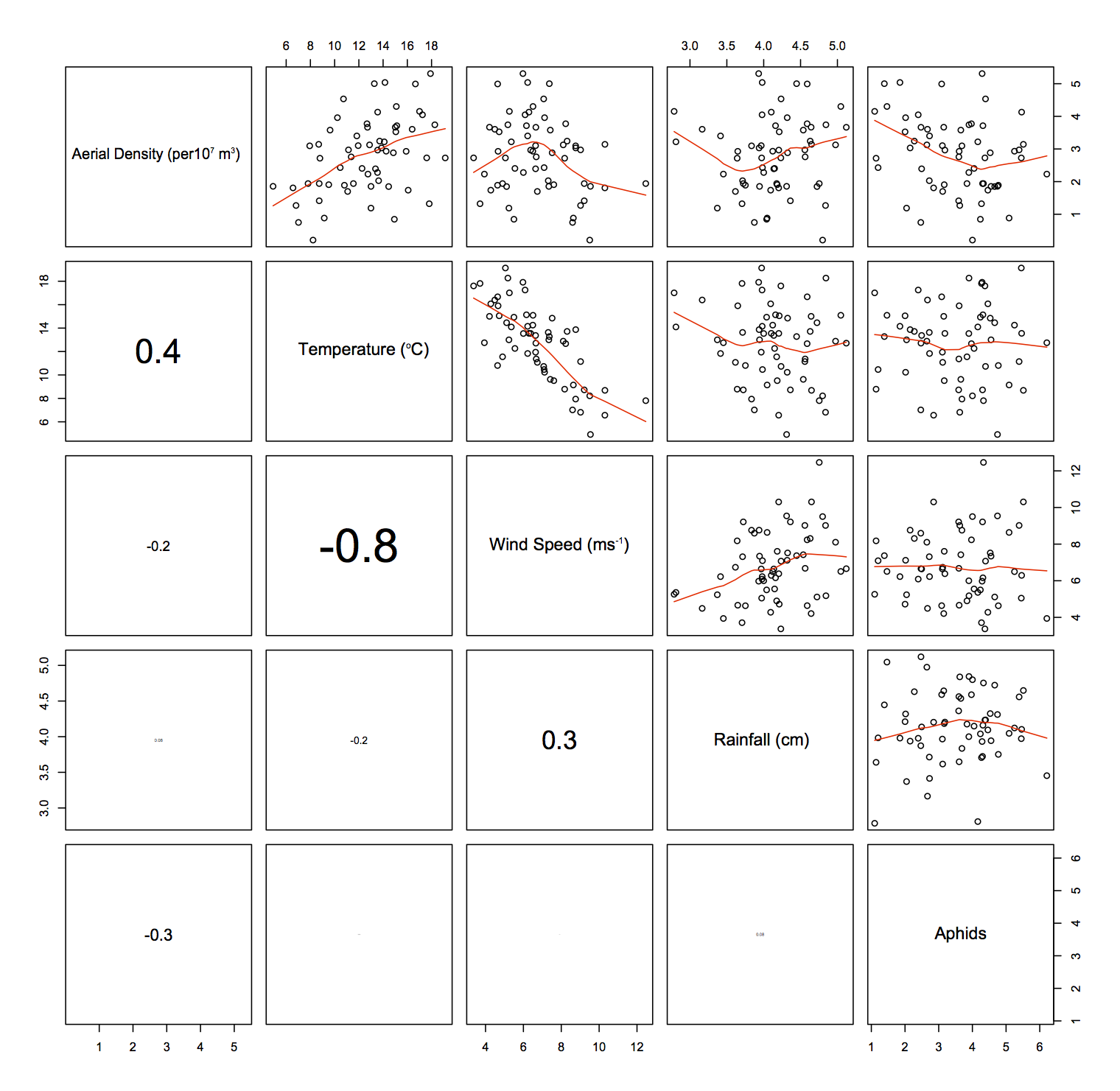

Supplement: Figure S4 — Pairplot of aerial density and explanatory variables. The lower diagonal panels contain the absolute correlations, with the font size proportional to the value. The upper diagonal shows the pair-wise scatter plots with LOESS smoothing lines added. Note aerial density, rainfall and aphids are log transformed. (TIFF) [file pone.0082278.s004.tiff]

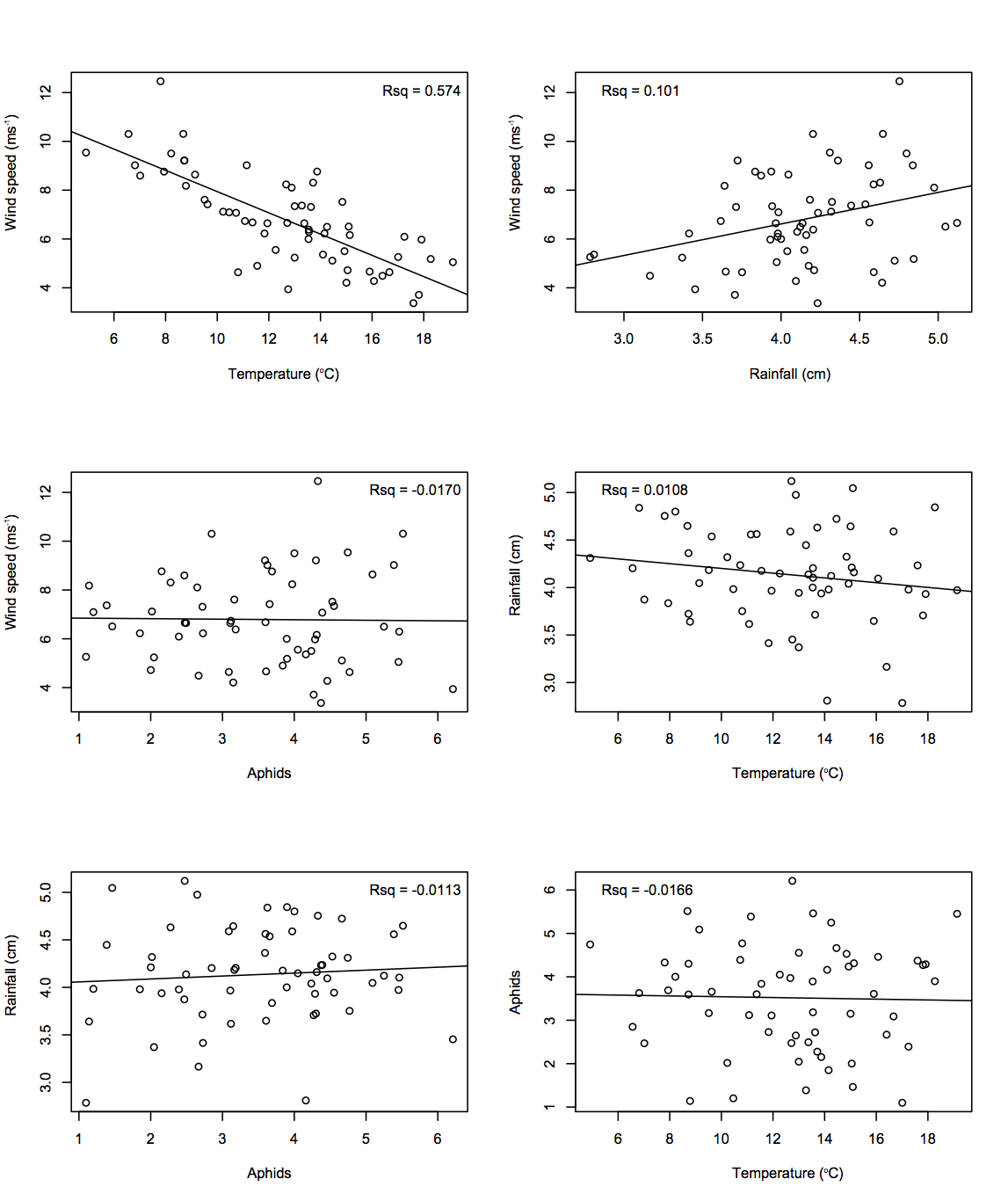

Supplement: Figure S5 — Relationship between explanatory variables. Pair-wise linear regression between explanatory variables. Note rainfall and aphids are log transformed. “Rsq” = R2adj (adjusted R2). (TIFF) [file pone.0082278.s005.tiff]

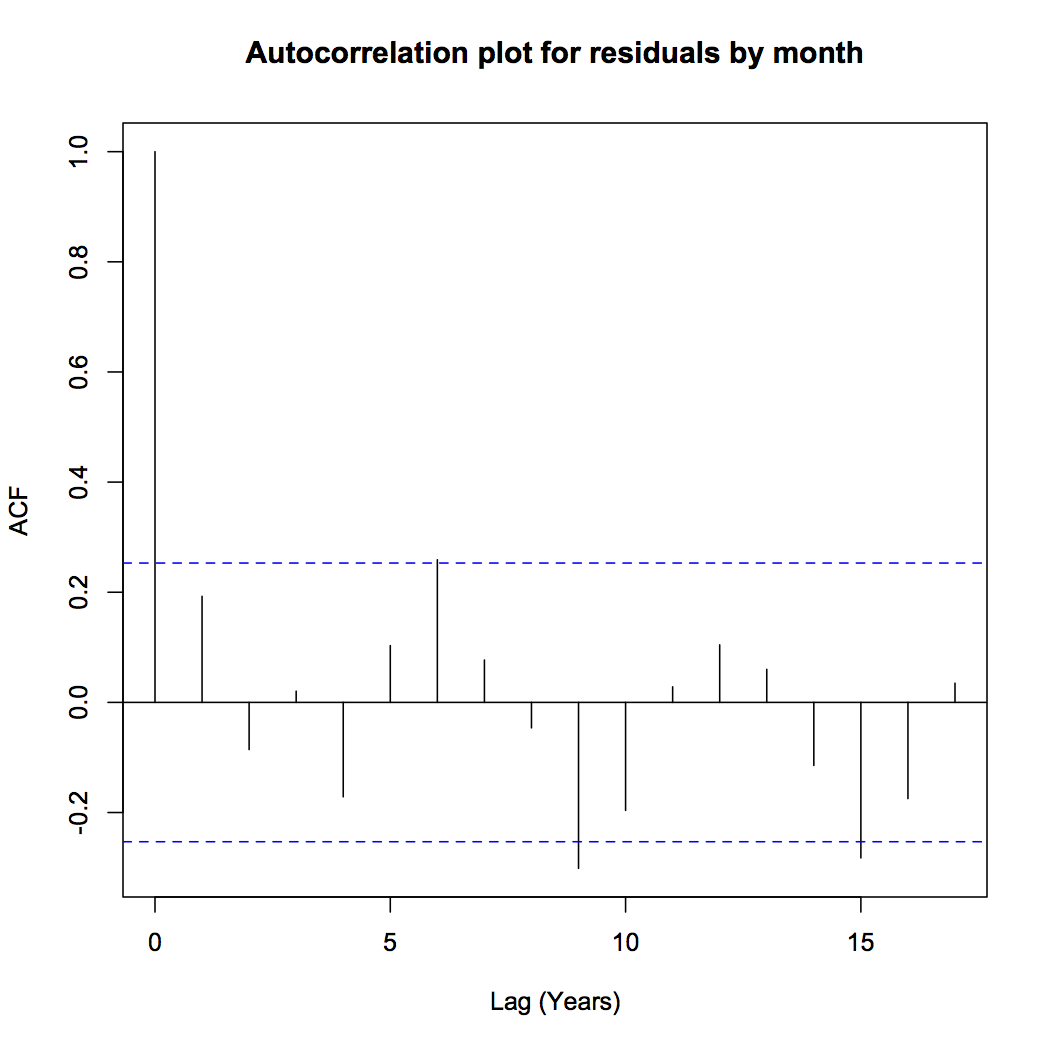

Supplement: Figure S6 — Auto-correlation plot of residuals for the selected minimal model (which includes the explanatory variables temperature and aphid abundance). “ACF” is the auto-correlation function. Peaks that cross the dotted blue lines are considered significant at the 5% level. The plot demonstrates marginally significant positive auto-correlation between the same month in different years, and negative auto-correlation at a lag of 9 and 15, which corresponds to 15 and 27-month intervals in real-time (e.g. between May and August, or June and September of different years). (TIFF) [file pone.0082278.s006.tiff]
