# Supplementary material for: Characteristics and Drivers of High-Altitude Ladybird Flight: Insights from Vertical-Looking Entomological Radar
Source: PLoS One. 2013 Dec 18;8(12):e82278. doi: 10.1371/journal.pone.0082278 (PMC3867359; doi:10.1371/journal.pone.0082278)
Supplement: Table S1 — Length ranges and number of records for all large ladybird species in the U.K. ladybird survey between 1990 – 2010. (DOCX) [file pone.0082278.s007.docx]

**Table S1. Length ranges and number of records for all large ladybird species in the U.K. ladybird survey between 1990 – 2010.**

| **Species name** | **Length (mm)** | **Number of records** | **% of total records (8 species)** |  |
| --- | --- | --- | --- | --- |
| 7-spot, *Coccinella septempunctata* | 5-8 | 27166 | 44.8% |  |
| Harlequin, *Harmonia axyridis* | 5-8 | 25676 | 42.3% **87.1%** |  |
| Orange, *Halyzia 16-guttata* | 4.5-6 | 4687 | 7.7% |  |
| Eyed, *Anatis ocellata* | 7-8.5 | 1425 | 2.3% |  |
| Cream-streaked, *Harmonia 4-punctata* | 5-6 | 1009 | 1.7% |  |
| Striped, *Myzia oblongoguttata* | 6-8 | 397 | 0.7% |  |
| Scarce 7-spot, *Coccinella magnifica* | 6-8 | 177 | 0.3% |  |
| Bryony, *Henosepilachna argus* | 5-7 | 146 | 0.2% |  |
| TOTAL |  | 60683 | 100.0% |  |
