# Supplementary material for: Characteristics and Drivers of High-Altitude Ladybird Flight: Insights from Vertical-Looking Entomological Radar
Source: PLoS One. 2013 Dec 18;8(12):e82278. doi: 10.1371/journal.pone.0082278 (PMC3867359; doi:10.1371/journal.pone.0082278)
Supplement: Table S2 — The proportion of the total number of aphid catches for all species detected by the Rothamsted Insect Survey (RIS) UK suction trap network that is accounted for by the 21 aphid species included in the RIS aphid bulletin. (DOCX) [file pone.0082278.s008.docx]

**Table S2.** The proportion of the total number of aphid catches for all species detected by the Rothamsted Insect Survey (RIS) UK suction trap network that is accounted for by the 21 aphid species included in the RIS aphid bulletin.

| **Bulletin species** | **Total Network catch** | **% of total** |
| --- | --- | --- |
| *Acyrthosiphon pisum* | 134833 | 1.25 |
| *Aphis fabae gp.* | 199274 | 1.85 |
| *Aulacorthum solani* | 9301 | 0.09 |
| *Brachycaudus helichrysi* | 386248 | 3.59 |
| *Brevicoryne brassicae* | 176603 | 1.64 |
| *Cavariella aegopodii* | 203214 | 1.89 |
| *Drepanosiphum platanoidis* | 707373 | 6.57 |
| *Elatobium abietinum* | 75531 | 0.70 |
| *Hyalopterus pruni* | 259627 | 2.41 |
| *Hyperomyzus lactucae* | 31698 | 0.29 |
| *Macrosiphum euphorbiae* | 49559 | 0.46 |
| *Metopolophium dirhodum* | 961280 | 8.93 |
| *Myzus (Nectarosiphon) ascalonicus* | 23618 | 0.22 |
| *Myzus (Nectarosiphon) persicae* | 130001 | 1.21 |
| *Nasonovia ribisnigri* | 6522 | 0.06 |
| *Phorodon humuli* | 253330 | 2.35 |
| *Rhopalosiphum insertum* | 1489866 | 13.85 |
| *Rhopalosiphum maidis* | 14417 | 0.13 |
| *Rhopalosiphum padi* | 2871907 | 26.69 |
| *Sitobion avenae* | 1314127 | 12.21 |
| *Sitobion fragariae* | 86171 | 0.80 |
| Bulletin Total | **9384500** | **87.22** |
|  |  |  |
| Remaining network Spp. (n = 149) | 1375667 | 12.78 |
