# Supplementary material for: Characteristics and Drivers of High-Altitude Ladybird Flight: Insights from Vertical-Looking Entomological Radar
Source: PLoS One. 2013 Dec 18;8(12):e82278. doi: 10.1371/journal.pone.0082278 (PMC3867359; doi:10.1371/journal.pone.0082278)
Supplement: Table S3 — Summary of the number of target species VLR records and aerial density by year and month. The total number of target species records in the VLR database is 8935. “S.D.” Standard deviation. (DOCX) [file pone.0082278.s009.docx]

**Table S3:** **Summary of the number of target species VLR records and aerial density by year and month**

The total number of target species records in the VLR database is 8935. “S.D.” Standard deviation

|  | **Number of Records** |  | **Aerial Density** |  |
| --- | --- | --- | --- | --- |
|  | **Sum** | **Mean (S.D.)** | **Sum** | **Mean (S.D.)** |
| **Year** |  |  |  |  |
| 2000 | 438 | 73.000(88.007) | 3013.916 | 502.319(587.431) |
| 2001 | 842 | 140.333(172.104) | 6284.291 | 1047.382(1362.642) |
| 2002 | 597 | 99.500(71.902) | 4001.248 | 666.875(483.737) |
| 2003 | 648 | 108.000(109.072) | 4928.638 | 821.440(861.099) |
| 2004 | 976 | 162.667(172.896) | 6224.867 | 1037.478(1144.627) |
| 2005 | 961 | 160.167(258.212) | 7838.928 | 1306.488(2168.020) |
| 2006 | 1510 | 251.667(299.544) | 14316.485 | 2386.081(2968.930) |
| 2007 | 527 | 87.833(68.974) | 3620.390 | 603.398(476.583) |
| 2008 | 755 | 125.833(95.943) | 4946.965 | 824.494(582.824) |
| 2009 | 676 | 112.667(95.261) | 5149.743 | 858.291(737.295) |
| 2010 | 1005 | 167.500(293.085) | 6483.900 | 1813.650(1813.263) |
| **Month** |  |  |  |  |
| May | 409 | 37.182(25.099) | 2767.449 | 251.586(179.903) |
| June | 939 | 85.364(55.276) | 6639.797 | 603.618(403.234) |
| July | 2965 | 269.545(192.169) | 22021.747 | 2001.977(1872.020) |
| August | 3460 | 314.545(241.609) | 26751.151 | 2431.923(1924.172) |
| September | 1064 | 96.727(54.161) | 7841.424 | 712.857(425.795) |
| October | 98 | 8.909(6.332) | 787.803 | 71.618(63.854) |
