# Supplementary material for: Characteristics and Drivers of High-Altitude Ladybird Flight: Insights from Vertical-Looking Entomological Radar
Source: PLoS One. 2013 Dec 18;8(12):e82278. doi: 10.1371/journal.pone.0082278 (PMC3867359; doi:10.1371/journal.pone.0082278)
Supplement: Table S4 — Summary of the number of target species VLR records, aerial density and displacement speed by altitude. (DOCX) [file pone.0082278.s010.docx]

**Table S4: Summary of the number of target species VLR records, aerial density and displacement speed by altitude**

| **Range Gate Number** | **Altitude** | **Number of records** | **Sum of aerial density** | **Percentage of total aerial density** | **Mean aerial density (S.D.)** | **Mean displacement speed (S.D.)** |
| --- | --- | --- | --- | --- | --- | --- |
| 1 | 150-195 | 1458 | 18144.572 | 27.159 | 12.445 (6.810) | 8.520 (4.153) |
| 2 | 221-266 | 1480 | 14162.332 | 21.198 | 9.569 (4.859) | 8.031 (3.344) |
| 3 | 292-337 | 1333 | 9864.980 | 14.766 | 7.401 (3.714) | 8.393 (3.393) |
| 4 | 363-408 | 1284 | 8266.099 | 12.373 | 6.438 (3.107) | 8.465 (3.307) |
| 5 | 434-479 | 1157 | 6509.547 | 9.743 | 5.626 (2.715) | 8.744 (3.243) |
| 6 | 505-550 | 776 | 3903.959 | 5.843 | 5.031 (2.423) | 9.295 (3.787) |
| 7 | 576-621 | 491 | 2283.039 | 3.417 | 4.650 (2.346) | 9.591 (3.746) |
| 8 | 647-692 | 383 | 1640.824 | 2.456 | 4.284 (2.012) | 9.974 (3.782) |
| 9 | 718-763 | 279 | 1088.765 | 1.630 | 3.902 (1.773) | 10.400 (3.748) |
| 10 | 789-834 | 159 | 550.473 | 0.824 | 3.462 (1.545) | 11.545 (3.830) |
| 11 | 860-905 | 84 | 261.351 | 0.391 | 3.111 (1.144) | 12.270 (3.425) |
| 12 | 931-976 | 38 | 98.934 | 0.148 | 2.604 (0.929) | 14.493 (3.945) |
| 13 | 1002-1047 | 11 | 30.858 | 0.046 | 2.805 (0.986) | 12.645 (4.198) |
| 14 | 1073-1118 | 2 | 4.047 | 0.006 | 2.024 (0.117) | 16.417 (1.307) |
| 15 | 1144-1189 | 0 | 0 | 0 | 0 | 0 |
