# Supplementary material for: Characteristics and Drivers of High-Altitude Ladybird Flight: Insights from Vertical-Looking Entomological Radar
Source: PLoS One. 2013 Dec 18;8(12):e82278. doi: 10.1371/journal.pone.0082278 (PMC3867359; doi:10.1371/journal.pone.0082278)
Supplement: Table S5 — Relationship between explanatory variables. Results of pair-wise linear regression exploring collinearity between explanatory variables. Upper diagonal = R2adj (adjusted R2), lower diagonal = F statistic and P value (in brackets). All F statistics are on 1 and 58 degrees of freedom. Significant relationships are highlighted in bold. Note rainfall & aphids are both transformed. (DOCX) [file pone.0082278.s011.docx]

**Table S5: Relationship between explanatory variables**

Results of pair-wise linear regression exploring collinearity between explanatory variables. Upper diagonal = *R^2^_adj_* (adjusted *R^2^*), lower diagonal = *F* statistic and *P* value (in brackets). All *F* statistics are on 1 and 58 degrees of freedom. Significant relationships are highlighted in bold. Note rainfall & aphids are both transformed.

|  | **Temperature** | **Wind speed** | **Rainfall** | **Aphids** |
| --- | --- | --- | --- | --- |
| **Temperature** | - | **0.5739** | 0.01078 | -0.01659 |
| **Wind speed** | **80.47 (1.484x10^-12^)** | - | **0.1014** | -0.01704 |
| **Rainfall** | 1.643 (0.205) | **7.654 (0.007586)** | - | -0.01126 |
| **Aphids** | 0.03689 (0.8484) | 0.01159 (0.9146) | 0.343 (0.5604) | - |
