# Supplementary material for: Characteristics and Drivers of High-Altitude Ladybird Flight: Insights from Vertical-Looking Entomological Radar
Source: PLoS One. 2013 Dec 18;8(12):e82278. doi: 10.1371/journal.pone.0082278 (PMC3867359; doi:10.1371/journal.pone.0082278)
Supplement: Table S6 — Drivers of high-altitude flight: full model (including wind speed). Results shown for qpGLMs and GLS are for full model containing all explanatory variables including wind speed. “qpGLM is quasi-Poisson full model. Both t statistic, and F statistic with corresponding and P values given. Dispersion parameter for qpGLM ρ = 0.369, and deviance 21.777 on 55 df. GLS without autocorrelation is equivalent to standard multiple linear regression. P value codes: *** P<0.000; ** P<0.001; * P<0.01; + P<0.05. (DOCX) [file pone.0082278.s012.docx]

**Table S6. Drivers of high-altitude flight**: **full model (including wind speed)**

Results shown for qpGLMs and GLS are for full model containing all explanatory variables including wind speed. “qpGLM is quasi-Poisson full model. Both *t* statistic, and *F* statistic with corresponding and *P* values given. Dispersion parameter for qpGLM *ρ* = 0.369, and deviance 21.777 on 55 df. GLS without autocorrelation is equivalent to standard multiple linear regression. *P* value codes: ^***^*P* < 0.000; ^**^*P* < 0.001;  ^*^*P* < 0.01; ^+^*P* < 0.05.

| **Variable** | **qpGLM**  ***t* (*P)*** | **qpGLM deviance, F value and P, all on 1 df)** | **GLS without auto-correlation**  ***t (P)* on 55 residual DF** | **GLS without auto-correlation including year**  ***t (P)* on 54 residual DF** | **GLS without auto-correlation including month**  ***t (P)* on 54 residual DF** | **GLS with auto-correlation *t (P)* on 55 residual DF** |
| --- | --- | --- | --- | --- | --- | --- |
| Rainfall | 1.109 (0.272) | 22.232 (*F =* 1.148, *P =* 0.289) | 1.114 (0.270) | 1.413 (0.163) | 1.124 (0.266) | 1.033 (0.306) |
| Wind speed | 0.970 (0.336) | 22.123 (*F* = 0.873, *P* = 0.354) | 1.013 (0.315) | 0.836 (0.407) | 1.182 (0.243) | 0.728 (0.470) |
| Aphid abundance | -2.314 (0.024^+^) | 23.750 (*F* = 4.982, *P* = 0.030^+^) | -2.319 (0.024^+^) | -1.845 (0.071) | -2.424 (0.019^+^) | -2.409 (0.019^+^) |
| Temperature | 3.408 (0.001^**^) | 26.046 (*F* = 10.782, *P* = 0.002^*^) | 3.444 (0.001^**^) | 3.910 (0.000^***^) | 3.500 (0.001^**^) | 3.115 (0.003^*^) |
| Date (year) | n/a | n/a | n/a | -1.758 (0.084) | n/a | n/a |
| Date (month) | n/a | n/a | n/a | n/a | -0.821 (0.415) | n/a |
| AIC | n/a | n/a | 189.647 | 192.519 | 194.171 | 190.143 |
| BIC | n/a | n/a | 201.691 | 206.442 | 208.094 | 204.194 |
| loglik | n/a | n/a | -88.823 | -89.260 | -90.085 | -88.071 |
