# Supplementary material for: Characteristics and Drivers of High-Altitude Ladybird Flight: Insights from Vertical-Looking Entomological Radar
Source: PLoS One. 2013 Dec 18;8(12):e82278. doi: 10.1371/journal.pone.0082278 (PMC3867359; doi:10.1371/journal.pone.0082278)
Supplement: Table S7 — Drivers of high-altitude flight: partial model (excluding wind speed). Dispersion parameter for qpGLM ρ = 0.367, and deviance 22.123 on 56 df. P value codes: *** P<0.000; ** P<0.001; * P<0.01; + P<0.05. (DOCX) [file pone.0082278.s013.docx]

**Table S7. Drivers of high-altitude flight**: **partial model (excluding wind speed)**

Dispersion parameter for qpGLM *ρ* = 0.367, and deviance 22.123 on 56 df. *P* value codes: ^***^*P* < 0.000; ^**^*P* < 0.001;  ^*^*P* < 0.01; ^+^*P* < 0.05.

| **Variable** | **qpGLM**  ***t* (*P)*** | **qpGLM deviance, F value and P, all on 1 df)** | **GLS without auto-correlation**  ***t (P)* on 56 residual DF** | **GLS without auto-correlation including year**  ***t (P)* on 55 residual DF** | **GLS without auto-correlation including month**  ***t (P)* on 55 residual DF** | **GLS with auto-correlation *t (P)* on 55 residual DF** |
| --- | --- | --- | --- | --- | --- | --- |
| Rainfall | 1.480 (0.144) | 22.932 (*F* = 2.048, *P =* 0.158) | 1.553 (0.126) | 1.831 (0.073) | 1.593 (0.117) | 1.305 (0.197) |
| Aphid abundance | -2.409 (0.019^+^) | 24.250 (*F* = 5.386, *P* = 0.024^+^) | -2.409 (0.019^+^) | -1.895 (0.063) | -2.454 (0.017^+^) | -2.488 (0.016^+^) |
| Temperature | 4.058 (0.000^***^) | 28.285 (*F* = 15.600, *P*  = 0.000^***^) | 4.129 (0.000^***^) | 4.503 (0.000^***^) | 3.992 (0.000^***^) | 3.836 (0.000^***^) |
| Date (year) | n/a | n/a | n/a | -1.865(0.068) | n/a | n/a |
| Date (month) | n/a | n/a | n/a | n/a | -0.545(0.588) | n/a |
| AIC | n/a | n/a | 186.153 | 188.672 | 191.114 | 186.087 |
| BIC | n/a | n/a | 196.280 | 200.716 | 203.158 | 198.239 |
| loglik | n/a | n/a | -88.077 | -88.336 | -89.557 | -87.043 |
