# Supplementary material for: Characteristics and Drivers of High-Altitude Ladybird Flight: Insights from Vertical-Looking Entomological Radar
Source: PLoS One. 2013 Dec 18;8(12):e82278. doi: 10.1371/journal.pone.0082278 (PMC3867359; doi:10.1371/journal.pone.0082278)
Supplement: Text S1 — Supplementary Methods and Results. (DOCX) [file pone.0082278.s014.docx]

**Characteristics and drivers of high-altitude ladybird flight: insights from vertical-looking entomological radar.**

Daniel L. Jeffries^1^; Jason Chapman^2,3^; Helen E. Roy^4^; Stuart Humphries^1^; Richard Harrington^2^; Peter M. J. Brown^5^; Lori-J. Lawson Handley^1*^

**Supplementary methods and results**

***Vertical-looking radar (VLR) data and target species identification***

Radar equipment, mode of operation, and analysis capabilities are described in full elsewhere [1, 2-6]. Signals captured within the 15 altitude bands (Table S4) of the VLR are recorded for 5 minute periods once every 15 minutes, 24h/day, giving almost continuous coverage [1]. During the 10-min intervals between recording periods, signals are automatically analysed as described by Smith et al [3]. The returned signal is modulated in a way that is related to the size, shape, position and speed of the insect target. The target’s mass and shape is estimated from its radar scattering properties, along with its horizontal displacement speed, displacement direction, and body alignment [1, 4]. Mass and shape are considered species diagnostic characteristics [4] and were used to extract aerial density (AD) data for ‘large ladybird-type targets’, assumed to be *H. axyridis* and *C. septempunctata.* AD is expressed in terms of the mean number of insects per 10^7^ m^3^ as calculated for each 5-min sample period [1]. Identification of target species relies on two types of information within the radar signals: body mass, and ratios of radar scattering terms that depend on body shape (the maximum and minimum radar reflectivity, referred to as σ_xx_ and σ_yy_ respectively, [1, 4]. The ratio of σ_xx_ / σ_yy_ is expected to rise from 1:1 for perfectly spherical objects, to >>1 for long, thin-bodied insects. Twenty *H. axyridis* and ten *C. septempunctata* were collected from the field, frozen live for 10 minutes, weighed and then measured in a transmission line rig that produces measurements indicative of those given by the VLR (for full details see [4]). Calibration measurements were made using six steel spheres of known actual size, covering the possible range of the target species (0.0001cm^2^ – 1cm^2^). The backscatter (returned radar signal) values of the spheres were then plotted against their diameters to produce a calibration curve, which was used to translate the backscatter signals of *H. axyridis* and *C. septempunctata* measured in the transmission line rig into measurements of the insect’s actual radar cross section. Calibration measurements therefore identified a range of backscatter values for the target species, which were then used to filter the VLR database. The VLR data set was filtered to extract records for the two target species, with a σ_xx_ / σ_yy_ ratio between 1.5 and 4.1, and a mass range of 25-42 mg.

***Characteristics and drivers of high-altitude flight***

*Tethered flight experiments*

Tethered flight experiments were carried out in a custom-built 1m^3^ Perspex cube with steel frames, situated in a constant temperature room set at 18^o^C, which roughly corresponds to the optimum flight temperature for the study species (see main Results). Air movement within the cube was minimized using magnetic seals on the door, to avoid any influence on flight. The insects were tethered by tying fine fishing line (Maxima monofilament, 0.1 mm diameter) over the pronotum and forelegs. The tether, which was 35 cm long in total was then threaded through a 15 cm long clear Perspex tube fastened to the upper surface of the cube so that there was 20cm of free tether. A Panasonic SDR-S26 video camera, capable of 25 frames per second (fps), was suspended 0.7 m directly above the clear Perspex tube. Flight was recorded for up to 2 hours after “release”. Flight videos were analysed in ImageJ [7] using the plug-in  “Particle Tracker” [8] to tag and automatically track the insect frame by frame, and the time spent in active flight recorded. A total of 20 *H. axyridis* individuals, collected from Hull, U.K. in May 2010 were used in the experiment.

*Data exploration and model validation*

The normality assumption was checked by examining Quantile-Quantile (Q-Q) plots (not shown), and using Shapiro-Wilk normality tests, while homogeneity was explored by examining the spread of residuals in residuals versus fitted and scale location plots. Temperature and wind speed were normally distributed (temperature: *W* = 0.984, *P* = 0.603; wind: *W* = 0.976, *P* = 0.287) but the normality and homogeneity assumptions were violated for aerial density (*W* = 0.664, *P* = 1.934e-10), rainfall (*W* = 0.936, *P* = 0.003) and aphid abundance (*W* = 0.680, *P* = 3.848e-10), and these variables were therefore log transformed (after log transformation: aerial density: *W* = 0.987, *P* = 0.775; aphid abundance: *W* = 0.979, *P* = 0.400; rainfall: *W* = 0.976, *P* = 0.277). After log transformation there was no remaining pattern in the spread of residuals.

Colinearity between variables was examined using pairs plots (Figure S4) and linear regression (Figure S5 and Table S5). There is a strong, negative linear relationship between temperature and wind speed (correlation coefficient = -0.8, *R^2^_adj_ =* 0.574, *F_1,58_  =* 80.470, *P =* 1.484x10^-12^) and a positive linear relationship between wind speed and rainfall (correlation coefficient = 0.3, *R^2^_adj_ =* 0.101, *F_1,58_  =* 7.654, *P =* 0.008). Partial models, excluding wind speed were therefore constructed and compared to full models to examine the effect of colinearity between wind speed and other environmental variables. Note however that there was no evidence for significant interaction between any explanatory variables, including temperature and wind speed (*t* -1.563, *P* 0.125) in the full quasi-Poisson GLM.

The dispersion parameter for the GLMs was *ρ* < 1, residual deviance ranged from 21.777 on 55 df for the full model to 22.932 on 57 df for the minimal model, and any patterns in the residuals were eliminated by using a qpGLM, indicating that any potential problems of over dispersion had been overcome and the minimal model is valid.

Auto-correlation was investigated by examining the value of the auto-correlation function (ACF) at different time lags in auto-correlation plots of the residuals obtained for selected models. The auto-correlation function (ACF) is essentially the Pearson correlation coefficient of the time series with itself, after applying a lag of *k,* while the partial ACF removes any effect of correlation between successive points in the time series [9]. The auto-correlation plot (Figure S6) demonstrates marginally significant positive auto-correlation between the same month in different years, and negative auto-correlation at a lag of 9 and 15, which corresponds to 15 and 27-month intervals in real-time (e.g. between May and August, or June and September of different years).

Finally, the data were explored for any extreme, influential observations by examining Cook’s distance plots. No significant outliers were identified.

*Drivers of high-altitude flight*

*Removing wind speed from the full models*

Removing wind speed from the full models increased the significance of temperature in both the qpGLM and GLS models but did not affect the outcome for aphids (Tables S6 and S7). Removing wind speed also improved the GLS models slightly by decreasing their AIC, BIC and log likelihood scores (Tables S6 and S7).

*Adding date or an auto-correlation structure to the GLS models*

Neither year nor month were significant predictors in the full or partial GLS models (Tables S6 and S7). Adding date (either year or month) as an extra explanatory variable in the full or partial GLS models without auto-correlation increased AIC, BIC and log likelihood scores, and therefore did not improve the basic GLS models (Tables S6 and S7). Scores were marginally better for the models including year rather than month (Tables S6 and S7). Adding year to the models reduced the effect of aphid abundance to not significant at the *P* < 0.05 level, but had no effect on the significance of other explanatory variables. Replacing month with year had no effect on any of the explanatory variables.

Adding an auto-correlation structure to the basic GLS without auto-correlation (i.e. without year or month) had no effect on the significance of any of the explanatory variables in the partial model (Table S7) but slightly reduced the significance of temperature in the full and minimal models (Tables S6 and main text Table 1). Adding auto-correlation to the full GLS model marginally increased AIC, BIC and log likelihood scores and therefore did not improve the model (Table S6), while in the partial model it had no effect on AIC, marginally increased BIC, and decreased log likelihood (Table S7).

**References**

1. Chapman JW, Reynolds DR, Smith AD (2003) Vertical-Looking Radar: A new tool for monitoring high-altitude insect migration. BioScience 53: 503.

2. Chapman J, Smith AD, Woiwod I, Reynolds D, Riley J (2002) Development of Vertical-Looking Radar technology for monitoring insect migration. Computers and Electronics in Agriculture 35: 95–110.

3. Smith AD, Riley JR, Gregory RD (1993) A method for routine monitoring of the aerial migration of insects by using a Vertical-Looking Radar. Philosophical Transactions of the Royal Society of London Series B: Biological Sciences 340 : 393–404.

4. Smith AD, Reynolds DR, Riley JR (2000) The use of vertical-looking radar to continuously monitor the insect fauna flying at altitude over southern England. Bulletin of Entomological Research 90: 265–277.

5. Riley JR, Smith AD (1996) Signal processing in a novel radar system for monitoring insect migration. Computers and Electronics in Agriculture 15: 267–278.

6. Riley JR (1985) Radar cross section of insects. Proceedings of the Institute of Electrical and Electronics Engineers 73: 228–232.

7. Abràmoff MD, Magalhães PJ, Ram SJ (2004). Image Processing with ImageJ. Biophotonics International, July 2004

8. Sbalzarini IF, Koumoutsakos P (2005) Feature point tracking and trajectory analysis for video imaging in cell biology. Journal of Structural Biology, 151: 182-195.

9. Zuur AF, Ieno EN (2007) Analysing Ecological Data. New York. Springer.
